# E-education for medical Students using WSI in Egypt

**DOI:** 10.1186/1746-1596-8-S1-S39

**Published:** 2013-09-30

**Authors:** Essam Ayad

**Affiliations:** 1 Departament of Pathology. Faculty of Medicine, Cairo University & Italian Hospital in Cairo, Egypt

## Introduction

**Figure 1:**
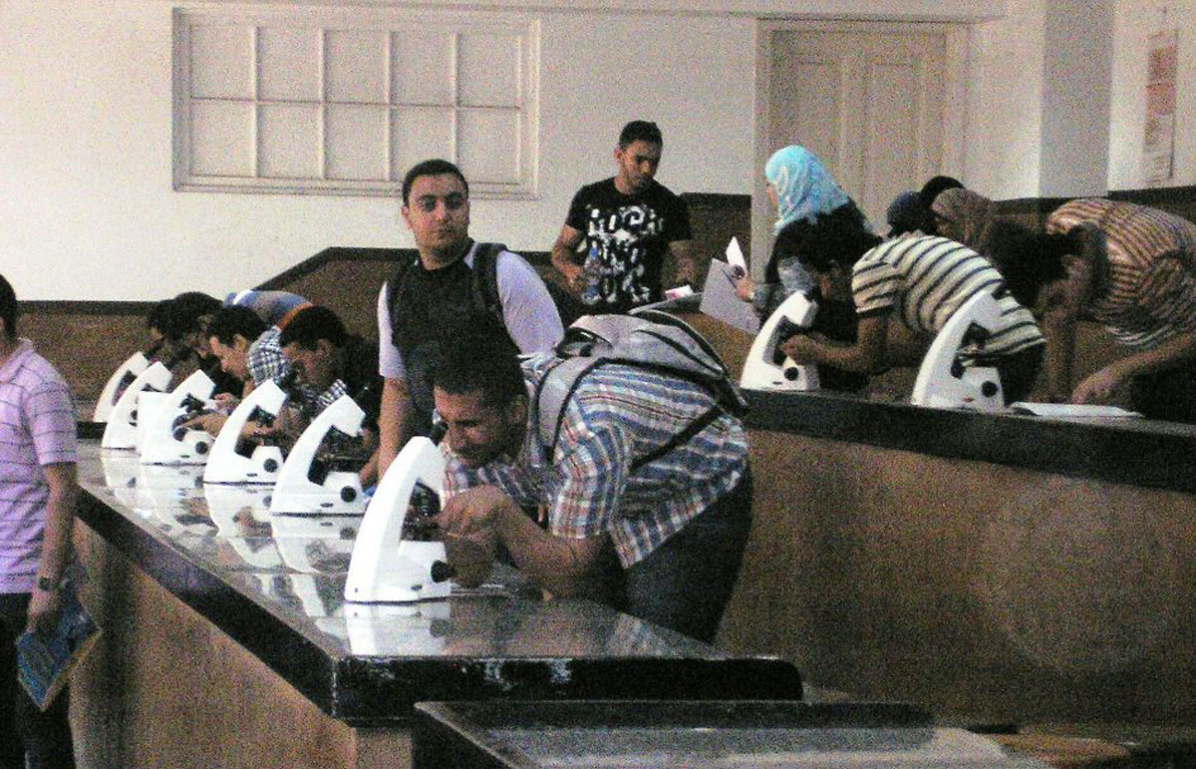
showed more than one student may have to share a microscope in the classic teaching laboratory

Classic practical Teaching of pathology for both under & post graduates requires microscopes and glass slides. Medical schools in developing countries are confronted with many challenges, among them, the large number of the students especially if this is compared with the limited resources of the medical schools. Furthermore, the high cost of acquisition and maintenance of microscopes. As a consequence, more than one student may have to share a microscope in the teaching laboratory (Figure 1).

Furthermore, glass slide specimens may also be shared among students since preparation of new specimens and replacement of broken slides are often expensive as well. Moreover, the time for laboratory hours is limited, and micro-scopes are often locked up and unavailable for use after class hours.

Although the medical schools in developed countries do not suffer a lot from the same problems but the reported trial for applying digital pathology techniques has shown to provide advantages of this digital technique over the usual method of teaching histology and pathology [1,2]. Few developing countries applied the digital pathology in the form of telepathology in clinical practice [3,4]. The use of digital pathology telepathology in education [5], second-opinion consultations [6] and primary diagnosis [7] has been reported in several journals. The early trials for applying digital pathology in medical education using the older techniques like the static & dynamic forms were not greatly satisfactory for the teachers or the students. Using the WSI technology changed the teaching circumstances completely. The entire microscopic glass slides were scanned and changed to digital files available on the Web server that can be viewed on a computer monitor with a Web browser. The dynamic and interactive mode of viewing images (horizontal and vertical movement of images, and zooming in and out) simulates viewing a glass slide under the microscope (virtual microscope). Its interactive features and facility to view the image on a large monitor promotes group interaction and discussion. Images can be viewed anytime with an Internet or Intranet connected desktop computer, portable or tablet computer, or even through smartphones anywhere. Its main challenge may be the requirement for high bandwidth. Implementing digital pathology in low-resource areas remains a challenge even today. Access to the Internet on academic networks is often slow and expensive. Other barriers include: the high cost of equipment for digitizing glass slides and limited student access to computer workstations especially after class hours. Very few trials tried to explore the student perspective of digital pathology especially in developing countries [8]. In this paper, we attempt to determine the student’s attitudes in one medical school towards digital pathology.

## Methods

Teaching glass slides were provided by the Department of Pathology, Cairo University. The whole set of slides were scanned using Bioimagene iScan 2, then the JP2 files gained were uploaded on the computer network in the pathology department computer lab and the Grand Student Library in the Faculty of Medicine, Cairo University. The images were configured for display on Apple devices (iPhone, iPod and iPad) using a secure PIN given for the students included in this pilot trial.

**Figure 2:**
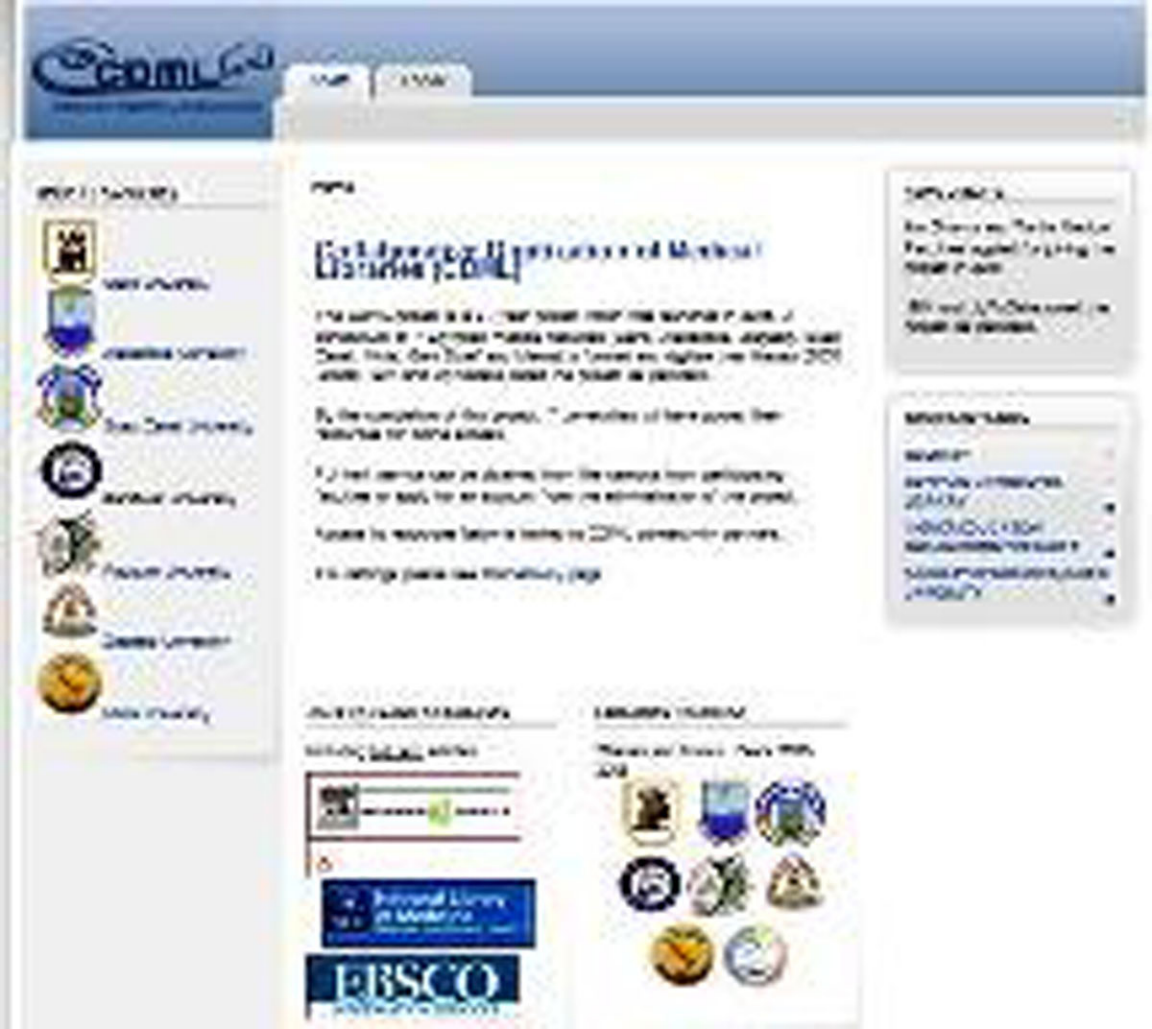
showed the Faculty severer website

Thirty five medical students (male = 29, female = 6; age range, 20–22 years, mode = 21 years) were selected as one group of practical lessons. After verbal consent was obtained, they were instructed to view both classic practical pathology teaching course using the glass slides and another digital teaching using the virtual slides available in the Pathology department computer network and that uploaded on the Faculty severer. Figure (2)

**Figure 3:**
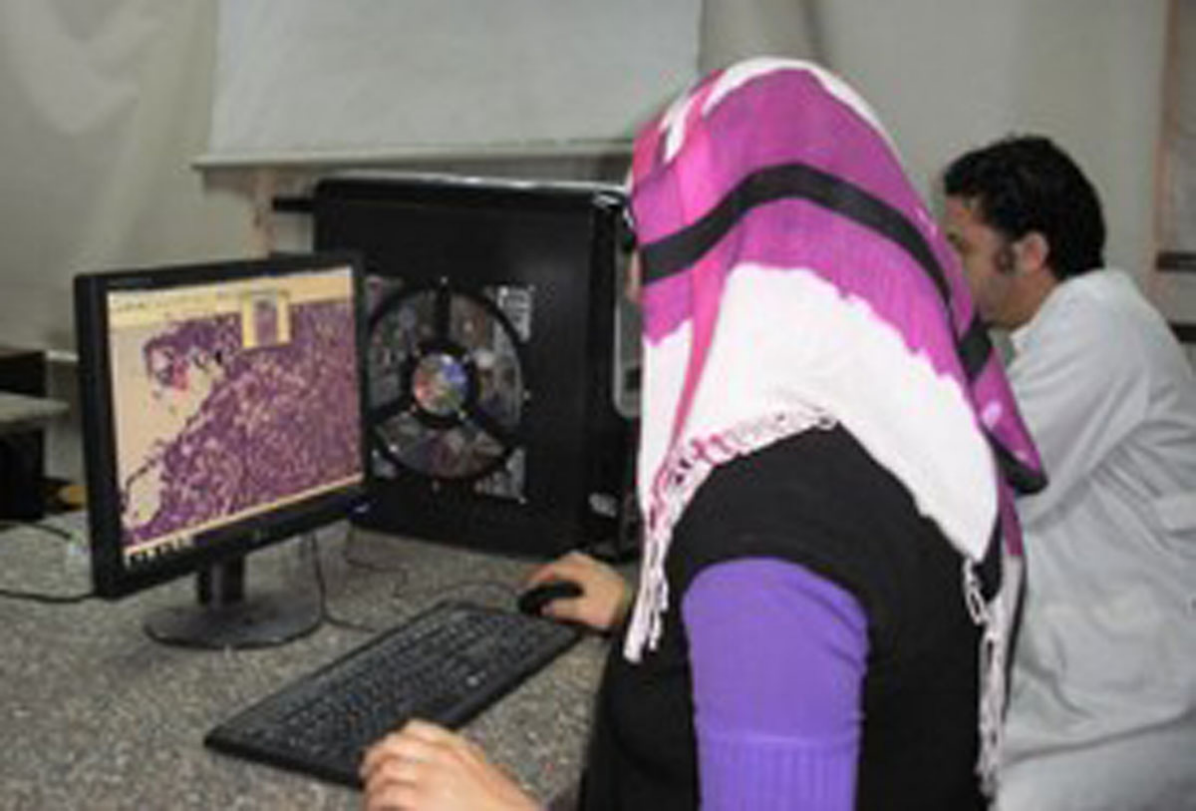
showed a participating student in the digital exam

The participants used a first generation iPad (Apple) tablet computer and get connected to the medical school’s shared wireless network with average tested speeds of 120 Kilobits/sec download and 41 Kilobits/sec upload. The Website was configured for devices that did not require Adobe Flash player; furthermore the image browsing program was also uploaded so there was no need to download any additional programs to see the images. By the end of the digital pathology teaching course, the students were asked to complete a paper-based evaluation. They reported their acceptance of the trial and rated their experience based on their perception of ease of navigation, access speed, and preferences comparing the digitized images seen in the department computer network and that seen from Web server through the wireless access in the faculty library or at home using the internet accessibility in a scale (1-Strongly Disagree, 2-Disagree, 3-Somewhat agree, 4-agree, 5-Strongly Agree). Comments were encouraged but not required. No personally identifiable information was collected. The students finally passed two practical exams; a routine exam using the microscope glass slides & another digital exam using the computer lab in the department uploaded with the virtual slides Figure [3].

The grades of both exams were collected and statistically analyzed & compared. Furthermore, we compared both grades [classic & digital] of this pilot group with the results of the rest of the student who received only classic glass-slides practical teaching.

## Results

More than 90% of the participating medical students rated the local computer network as significantly faster and responsive to their school needs. The participants also evidently preferred using the wireless connection in the library rather than reaching the website at home using the internet. Although not required, all of the students provided written comments to almost all of the questions posed. Their observations generally revealed that the images from the local server loaded faster and of the almost seamless quality in viewing the slides displayed. They also validated that virtual slides simulate the experience of examining glass slides under an optical microscope. Some general comments common to both servers were: “dealing with the pictures on the computer screen was much easier than the glass slides and the microscope”, “interactive”, “pathology studies became much easier & interesting”, “wonderful quality pictures,. The slides were labeled with the diagnosis and the description of the main diagnostic features but all these data could be seen when needed. Concerning the specific comments on the servers partially reflect the bandwidth deficiency that slowed the display of digital images from the server. They found the local server to be “fast loading & zooming in local server”.

**Table 1:** showed the comparison between the student’s grades in both classic & digital practical exams. The results indicated the better understanding of the practically taught material through the digital form rather the classic one.

	Classic	Digital
Number of students	35	35

Range [out of 10]	3-10	6-10

Mean	8.1	8.9

Percentage	81	89

**Table 2:** showed the comparison between the trial group grades [classic & digital] and the grades of the rest of the student who received only classic glass-slides practical teaching.

	Trial Group	Rest of Students
Number of students	35	140

Range [out of 10]	3-10	3-10

Mean	8.1	7.33

Percentage	81	73.3

These results reflected that the understanding of the practical pathology material learned [by microscopes & classic glass slides] was evidently better & easier for student received digital teaching also as the students reported in their comments about the clarity of the images and how was it easy to navigate through the virtual slides.

## Discussion

Although Internet users in Egypt are more than 35% of the population, the bandwidth in most places is still less than 2 Mb/s. In our study, the network in the medical school, which is situated at the computer network in the pathology department or the Grand Student Library in the Faculty of Medicine, Cairo University is rated at 8 Mb/s. Although the medical students found their experience to be generally satisfactory in this study, digital pathology implementation in developing countries may still be limited by slow connections to the Internet [8]. However, we believe that the slow connectivity or unavailability of Internet access will improve soon in most of the developing courtiers so it need not hamper the use of digital pathology in these countries. The increasing capacity and lowering costs of these portable storage devices make them suitable alternatives to accessing virtual slides through the Internet. The use of digital slides in medical education makes the study of pathology a meaningful yet interesting endeavor for students [10]. In this study, we received positive comments that showed how students perceived the use of digital pathology in medical school. The students also suggested that the option to switch between labeled and unlabeled versions was useful and easier to navigate and reaching the diagnosis. This is a feature that has been suggested in the past through a previous trial [8]. In developed countries, local area networks are often assumed to be robust and adequately managed. However, it does not usually prevail in low-to-medium income countries where reliability is a continuing challenge. On top of the expense of network hardware, there is also the cost of hiring and retaining competent technical personnel to install, configure, and keep the local network running at optimal levels.

Although local access to the digital slides received better reviews from the students in this study, the hidden cost of keeping a reliable network should be considered when planning the appropriate placement of servers and wireless networks. Since the study was done in a single medical school in Egypt, we hope that it can be universally applicable. However, since many of the conditions mentioned earlier prevail in developing countries, our study highly encourage the suggestions on the implementation of digital pathology in low-resource areas. Estimation of the minimum acceptable bandwidth for viewing virtual slides needs further studies. The cost of scanning slides is still prohibitive in most developing countries. Collaboration between academic centers in developed countries with digital scanning equipment and universities in developing countries with teaching slides that may be rare and unusual in developed countries, could enhance medical education for all [8]. Ultimately, it may be cost-effective for a university to send their teaching slides to partner institutions by mail or courier who can digitize them for sharing across participating schools.

## Conclusion

Our results show that access to the server either through the wireless connection in the computer network in the pathology department or the Grand Student Library in the Faculty of Medicine, Cairo University or through the internet at home was satisfactory but the local server was deemed faster and preferred by a majority of participants in this study. Virtual slides, accessible through a local server or portable drive may be a solution to the high bandwidth requirement of digital pathology or in places where the Internet in unavailable. Collaborations with universities in developed countries would enhance image collections for teaching for institutions and these can be shared with others.

## Competing interests

The author declares that they have no competing interests.
